# Current News

**Published:** 1896-08

**Authors:** 


### Current News.

### HARVARD DENTAL ALUMNI ASSOCIATION.

The silver anniversary and twenty-fifth annual banquet of the Harvard Dental Alumni Association was held at "The Thorndike," Boston, on June 22, 1896, with sixty-three members and guests present.

Reports of the secretary, treasurer, and committee on Harvard Dental School were submitted, showing the prosperous condition of the Association, etc.

The invited guests present were Rev. Reuen Thomas, D.D., Brookline; Hon. Harvey N. Shepard, Boston; Rev. Willard T. Perrin, S.T.B., Boston, and Mr. John H. Colby, ex-councilman of Boston city government.

The post-prandial exercises were inaugurated by President James Shepherd, who feelingly alluded to the deaths of Professor Thomas H. Chandler, dean of the Dental School, and Frederick H. Woodcock, instructor in mechanical dentistry. He then called upon the new dean, Professor Eugene H. Smith, as the first speaker. Dr. Smith, after eulogizing his predecessor and Dr. Woodcock, spoke of the wants of the school,—that greater facilities were needed for increasing the scope of its work. A new building was a necessity, now that chemistry was no longer to be taught in the medical school, and must be in the dental building; also that an endowment fund was required. The graduating class numbered twenty-two, the largest within its history, and though the requirements are yearly becoming severer, yet the number of students continue to increase. Each guest responded in a happy vein with an excellent speech, and in a few instances in a humorous manner.

The following named officers were elected for the ensuing year: President, Frank Perrin, D.M.D., 1877; Vice-President, Joseph T. Paul, D.M.D., 1891; Secretary, Waldo E. Boardman, D.M.D., 1886; Treasurer, Washburn E. Page, D.M.D., 1877.

*Executive Committee.*—Waldo E. Boadrman, D.M.D., chairman; William P. Cooke, D.M.D., and Harry S. Parsons, M.D., D.M.D.

The council is composed of the officers of the Association.

WALDO E. BOARDMAN, D.M.D.,
*Secretary.*

BOSTON, June 25, 1896.

